# Design and Evaluation of an Ultrahigh-Strength Coral Aggregate Concrete for Maritime and Reef Engineering

**DOI:** 10.3390/ma14195871

**Published:** 2021-10-07

**Authors:** Jinming Liu, Boyu Ju, Wei Xie, Huang Yu, Haiying Xiao, Shanliang Dong, Wenshu Yang

**Affiliations:** 1 School of Materials Science and Engineering, Harbin Institute of Technology, Harbin 150001, China; 2 Defense Engineering of Academy of Military Sciences, PLA Academy of Military Sciences, Beijing 100036, China; Liujm1025@outlook.com (J.L.); xieweixiongqi@163.com (W.X.); 3 School of Astronautics, Harbin Institute of Technology, Harbin 150001, China

**Keywords:** ultrahigh-strength marine concrete, self-compacting, compressive strength, optimal mixture design

## Abstract

In this paper, an ultrahigh-strength marine concrete containing coral aggregates is developed. Concrete fabricated from marine sources is considered an effective and economical alternative for marine engineering and the construction of remote islands. To protect sea coral ecosystems, the coral aggregates used for construction are only efflorescent coral debris. To achieve the expected mechanical performance from the studied concrete, an optimal mixture design is conducted to determine the optimal proportions of components, in order to optimize the compressive strength. The mechanical properties and the autogenous shrinkage, as well as the heat flow of early hydration reactions, are measured. The hydration products fill up the pores of coral aggregates, endowing our concrete with flowability and self-compacting ability. The phases in the marine concrete are identified via X-ray diffraction analysis. The 28-day compressive and flexural strength of the developed marine concrete achieve 116.76 MPa and 18.24 MPa, respectively. On account of the lower cement content and the internal curing provided by coral aggregates, the volume change resulting from autogenous shrinkage is only 63.11% of that of ordinary reactive powder concrete.

## 1. Introduction

The protection of marine reefs plays a great role in the development of island economies, as well as marine resources. The active demand of infrastructural construction on the islands accelerates the development of construction materials in the context of forming a conservation-minded economy and an environmentally sound ecosystem. Due to the limitation of the utilization of conventional construction materials in terms of time and transportation expenses, as well as the complex geological conditions of islands, challenges arise as to the production of alternative cementitious materials. The infrastructure on islands is situated in an aggressive marine context. They might be subjected to tidal waves or freeze/thaw actions, presenting more stern requirements on the load-carrying capacity of materials, as well as the durability and long-term stability of structures.

Concrete fabricated from marine sources (marine sand, seawater, efflorescent coral debris, etc.) is considered an effective and economical alternative. Coral-based concrete has been extensively used in building roads, airports, and other structures during and after the Second World War [1], especially on islands in the western Pacific Ocean [1,2,3,4,5]. The South China Sea possesses large deposits of weathered coral debris. The coral aggregates can be used to fabricate concrete when there are limitations in using conventional aggregates [6,7]. Research has demonstrated how coral concrete can be applied to the construction of breakwaters, sand dykes, bank revetments [8], retaining walls [9], pavements, and building foundations [10]. One such aggregate was even used in emergency repair for marine engineering [11].

**Figure 1:**
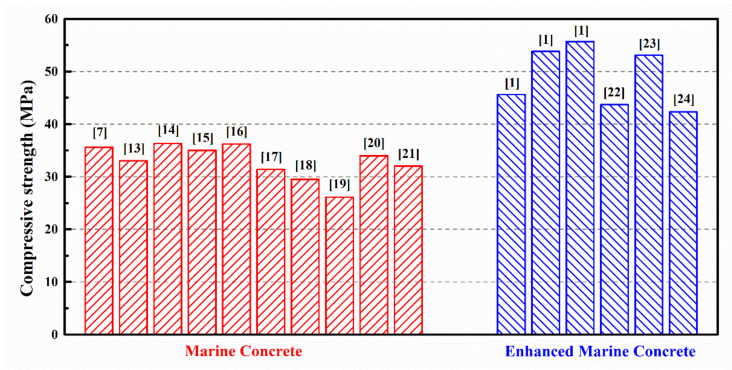
Compressive strength of marine concrete (30 MPa) [7,13,14,15,16,17,18,19,20,21] and enhanced marine concrete (40–50 MPa) [1,22,23,24].

Previous studies have shown that the original designed marine concrete has a relatively low compressive strength—approximately 30 MPa (Figure 1) [12,13,14,15,16,17,18,19,20,21]. The compressive strength of the marine concrete increases with the increase in the proportions of cementitious material and mineral admixture [22,23,24]. It can be observed (Figure 1) that the maximum compressive strength of 55.7 MPa was achieved with a designated water/binder ratio (W/B) and cement and mineral admixture contents (see Table 1). However, the construction of high-rise buildings [25], bridges [26,27], and protective structures [28,29] on the islands urges the development of high-strength construction materials to meet the requirements for the improvement of the design flexibility and the building functions.

The motivation of this study is to design a marine concrete with ultrahigh compressive strength to facilitate the development of marine and reef engineering. The main objective is to fabricate a coral-based concrete with a compressive strength of over 100 MPa under the marine environmental curing conditions. The densification of the studied concrete mainly counts on its self-compacting ability, rather than vibration in the mixing process. Meanwhile, the autogenous shrinkage is expected to be reduced by the autogenous curing provided by coral aggregates. The main ingredients used in the preparation of such concrete are conventional concrete gel materials, coral rock, coral sand, and seawater. The concrete mix proportions are defined by using the optimal mixture design method based on packing density theories [30] and the means of homogeneously arranged particles [31,32]. In this experiment, the mechanical properties, autogenous shrinkage, and heat of hydration are measured to quantify the performance of the designed concrete. The microstructures of the main constituents are identified in order to investigate how these microstructures affect the physical characterization of our concrete. Then, X-ray diffraction analysis is conducted to determine its chemical characterization.

This paper is structured as follows: In Section 2, the raw materials and experiments are introduced in the present study. The single-factor experiments are introduced in Section 3, and provide an estimated mix proportion for concrete. In Section 4, the optimal mix proportion is determined by mix designs, and this mix proportion is verified experimentally. The autogenous shrinkage and hydration heat of the optimized concrete are measured, and SEM and XRD analyses are provided to investigate the origination of the ultrahigh strength of our concrete. The concluding remarks are presented in Section 4.

## 2. Materials and Methods

### 2.1. Materials

In this study, the main raw materials used in the design of the ultrahigh-strength marine concrete (USMC) were ordinary Portland cement (OPC), silica fume (SF), cementitious capillary crystalline waterproof material (CCCW), superplasticizer (SP), coral sand, coral powder, and artificial seawater. The utilization of mineral admixtures such as silica fume as mineral additives was expected to enhance the engineering properties and the densification of the studied concrete, improving its mechanical performances [33,34].

#### 2.1.1. Cementitious System

The selection of materials was based on the Chinese National Standards associated with each material. The chemical constituents of the selected cement and silica fume agglomerations are listed in Table 2.

**Figure 2:**
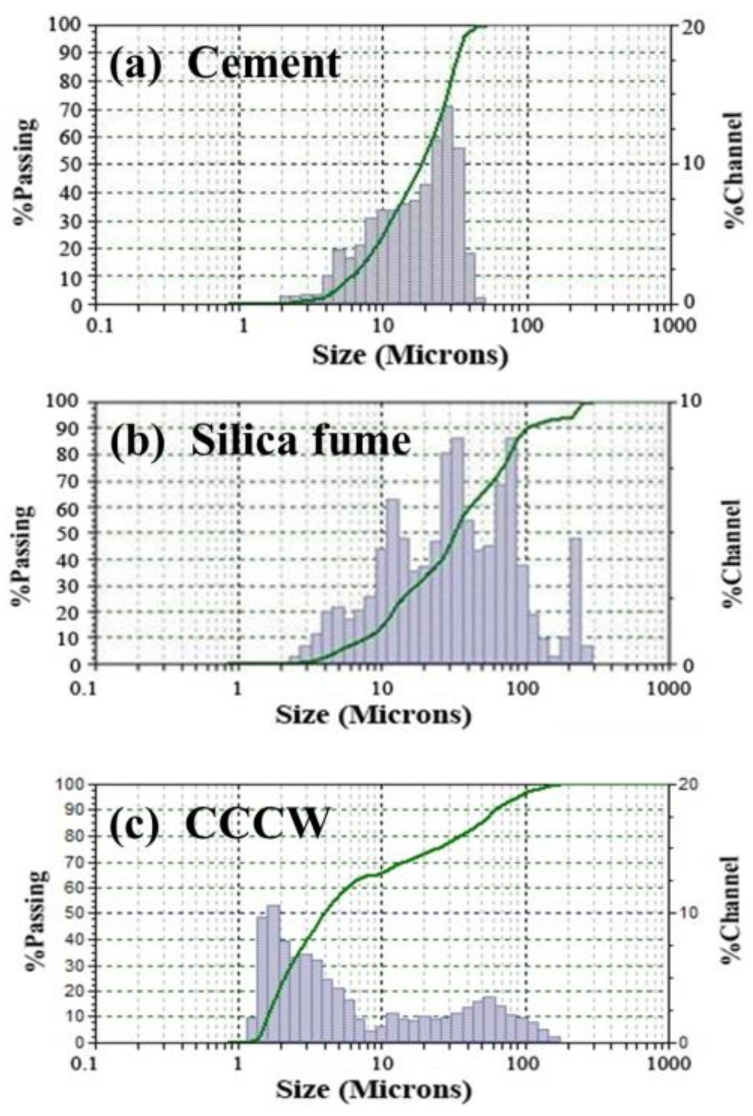
Particle-size distributions of (**a**) cement, (**b**) silica fume aggregate, and (**c**) CCCW.

Figure 2 shows the particle-size distributions of these two materials, where the particle-size distribution of the silica fume is between 0.1 μm and 0.3 μm. It is notable that due to the particle agglomeration in silica fume, the particle-size distribution shown in Figure 2b is inconsistent with the actual values reported by the manufacturer. CCCW compliant with Chinese National Standard GB 18445-2012 was employed in this research. The type of CCCW was “Yingzhi RS-7”, which was produced in the Rising Fine Chemical Factory. A polycarboxylate-based high-performance superplasticizer was added to the prepared samples to achieve the target workability, and the dosage in all samples was 2.4% of binder mass.

#### 2.1.2. Aggregates

For the sake of protection of the sea coral ecosystems, only efflorescent coral debris was extracted. The coral aggregates were used as fine granules for the preparation of the ultrahigh-strength concrete. Table 3 shows the determinations of the basic chemical and physical properties of the selected coral sand. Its main component was calcium carbonate, with a calcium content of up to 98%.

**Figure 3:**
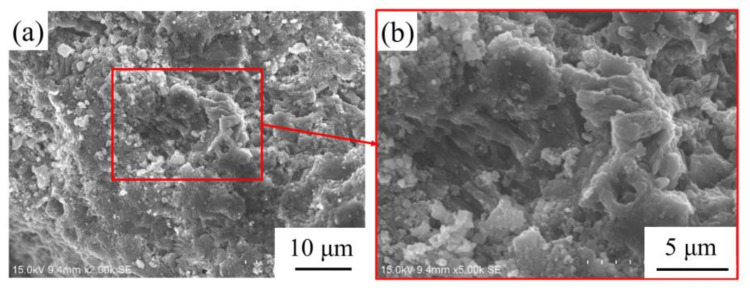
(**a**) Microstructures of coral sand, and (**b**) partial enlarged representation of the red box area in (**a**).

**Figure 4:**
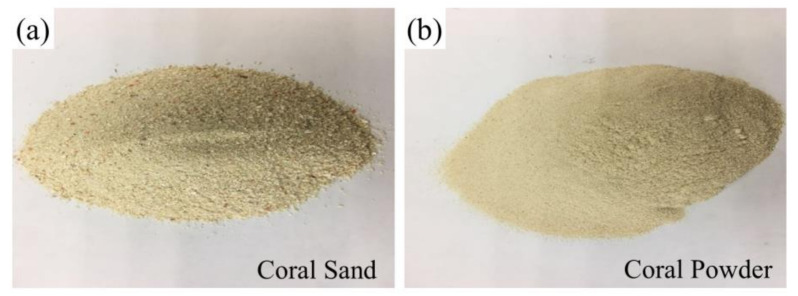
The macro picture of (**a**) coral sand and (**b**) coral powder.

The abundant pore structures as well as porous microstructures within the selected coral aggregates are shown in Figure 3. The coral sand and coral powder (Figure 4) were prepared via shattering and sieving processes. The coral sand with particle-size distribution of 0.6–1.18 mm and the coral powder of 0.075–0.3 mm were used as granular materials. The water absorption of the coral sand was 1.13%, and the water absorption of the coral powder was 2.97%.

#### 2.1.3. Artificial Seawater

The artificial seawater was composed of sea crystal and fresh water. A total of 50 kg of fresh water and 1.95 kg of sea crystal were mixed to achieve the average salinity of 35‰ of the world oceans.

### 2.2. Concrete Sample Preparation

To prepare the samples for experiments, a mixing procedure for the raw materials and the water mixture (mixture of the selected superplasticizer and artificial seawater) was performed in accordance with Chinese National Standard GB/T 31387-2015 “reactive powder concrete” [35], as follows:

(1) All dry-mixing powders (binder particles, coral sand, and coral powder) were stirred for 3 min;
(2) Water mixture was then added to the mixed dry materials, and the mixture was stirred for 5 min.

**Figure 5:**
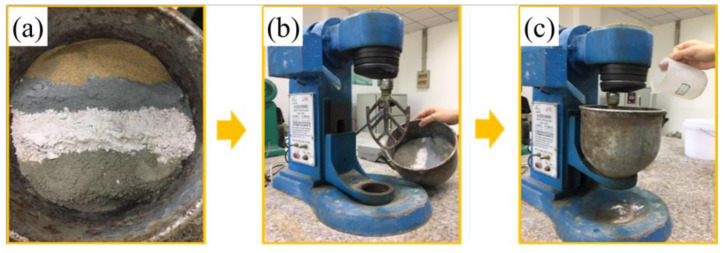
The process of preparing reactive powder concrete: (**a**) put the dry powder into the container; (**b**) stir the dry powder; (**c**) add water for further stirring.

The sequence of preparation is shown in Figure 5. The mixer used was a vertical cement mixer with a speed of 250 r/min.

### 2.3. Experimental Methods

#### 2.3.1. Strength Tests

All concrete samples were molded into cuboids with a geometry of 40 mm × 40 mm × 160 mm [36]. The specimens were cured in the artificial seawater for 27 days under natural conditions.

The strength tests of specimens were performed using a computer-controlled electro-hydraulic testing machine [37]. For the flexural strength test, the loading rate was set to 50 ± 10 N/s, while for the compressive strength test, the loading rate was 2.4 ± 0.2 kN/s.

In the property measurement, 3 samples were selected for testing each time. When the three data differed by no more than 15%, the average value was taken; if two of the three data differed by more than 15%, the data were invalidated and retested.

#### 2.3.2. Autogenous Shrinkage Test

**Figure 6:**
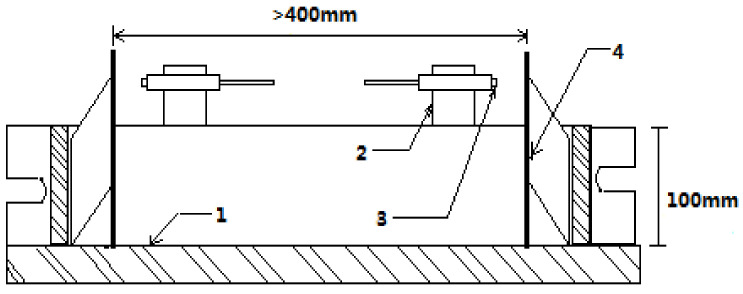
Non-contact concrete shrinkage deformation testing system (1—steel mold; 2—sensor support; 3—displacement sensor; 4—standard target).

The linear autogenous shrinkage of the designed concrete was measured using an eddy-current displacement sensor (ECDS) based on the electromagnetic induction effect [38]. Figure 6 illustrates the schematics of the test setup. The sequences of operation were as follows:

(1) The inner surfaces of the steel mold (100 mm × 100 mm × 550 mm) were smeared with grease. The Teflon sheets were placed to eliminate the friction between the inner surfaces of the steel mold and the concrete specimen;
(2) Two steel target seats were mounted on the proper positions of the bottom surface of the steel mold. In this study, the distance of the two target seats was 455 mm. Standard targets were magnetically attached to the steel target seats;
(3) The mixture was poured into the steel mold and encapsulated the steel target seats, guaranteeing that the target seats would deform simultaneously with the specimen. The thermocouples were embedded into the center of the specimen to isolate the autogenous shrinkage from the measured total deformation by monitoring the temperature changes within it;
(4) The top surface of the specimen was sealed with two layers of polyethylene sheets to avoid the influence of exterior drying. The sensor support was fastened to the top of the steel mold. By regulating the fitting screw, the ECDS was mounted on a proper position;
(5) In our measurements, the interval of data acquisition was 1 min;

During the entire testing period, the temperature was maintained at 20 ± 1 °C. The measured autogenous shrinkage was set to zero at the initial setting time determined by the corresponding pure concrete sample. The results of deformation were obtained by measuring three specimens in parallel to achieve a better estimation. Results were compared with reactive powder concrete and original mix proportion concrete [39].

#### 2.3.3. Hydration Heat

The autogenous shrinkage of samples increases with the concentrated and high hydration heat [40]. The hydration heat experiment was conducted to further analyze the autogenous shrinkage of samples, as well as to verify the results of the autogenous shrinkage tests. The hydration heat flows of samples were measured using an eight-channel TAM Air heat-conduction calorimeter (TAM, Thermometric AB Co. Ltd., Kista, Sweden). The thermogravimetric (TG) and differential scanning calorimetry (DSC) curves of the pure sample that came from the same batches in the TAM semi-adiabatic calorimeter were obtained by using a TA Q50 instrument. In order to minimize the influence of temperature increase caused by external operations, the hydration heat was measured 1 h after the mixing procedure. The testing took 72 h. During the testing, the operating temperature was kept at 20 °C. The results were normalized by the weight of the sample.

#### 2.3.4. Scanning Electron Micrograph

In order to compare the content and distribution of pores in the cement samples [41], the micrographs of samples were taken using a JSM-6490LV scanning electron microscope (Japan Electronics Co. Ltd., Tokyo, Japan). In order to image the surfaces accurately, the samples were sprayed with gold powder to increase their to conductivity. The voltage was 12 kV and the working distance was ~12 mm. The instrument was vacuumized for 10 min prior to testing.

#### 2.3.5. X-ray Powder Diffraction

X-ray powder diffraction was used for characterization of the coral sand and identification of the minerals in the specimens [42]. Data were collected from 5° to 80° at a scan rate of 4°/min. The angular step was 0.02°. The phases were analyzed using Jade 6.0 software.

## 3. Results and Discussion

### 3.1. The Effect of a Single Binder on Mechanical Properties

The single-factor experimental designs were performed to quantify the independent effects of the materials of the binder on the mechanical performance of the studied concrete. The single-factor designs prescreen and determine the range of each of the material contents, which can be beneficial to the enhancement of the mechanical strength of our concrete.

Via a series of preliminary experiments, the primary material constituents and mixing proportion were determined (Table 4) for the preparation of the designed ultrahigh-strength marine concrete.

**Figure 7:**
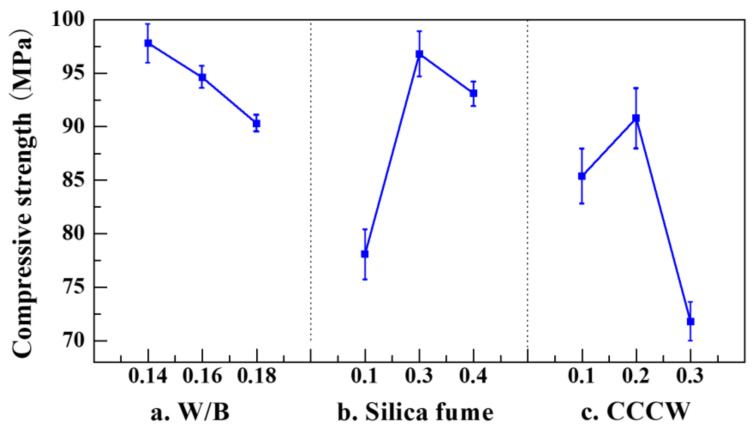
Compressive strength of USMC with different admixtures: (**a**) W/B, (**b**) Silica fume, (**c**) CCCW.

**Figure 8:**
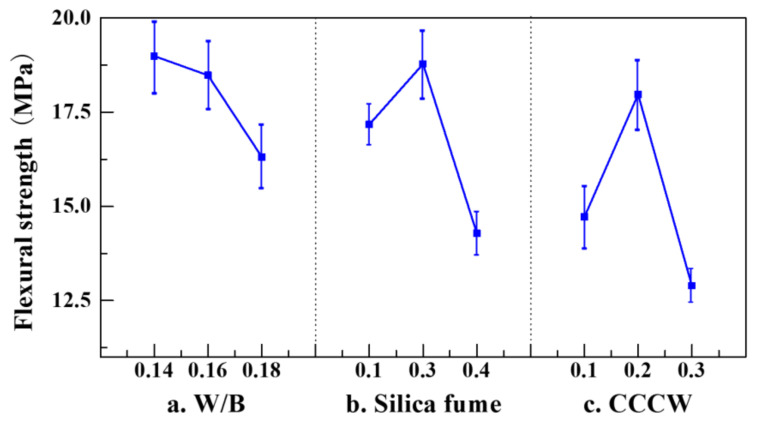
Flexural strength of USMC with different admixtures: (**a**) W/B, (**b**) Silica fume, (**c**) CCCW.

The mixing proportions prepared for single-factor experiments are given in Table 5. The measured compressive strength and flexural strength of each sample are shown in Figure 7 and Figure 8, respectively.

It can be observed from Figure 7and Figure 8 that both compressive strength and flexural strength monotonically decrease with an increase in W/B. A part of the water hydrates the cement, while the remaining free water forms bubbles or pores after the evaporation process [43]. These bubbles or pores reduce the homogeneity of the concrete [31]. Additionally, the stress concentration is observed around the pores under external loads [44]. The lower the W/B, the denser the concrete, and the higher its strength [31]. However, the low W/B (0.14) also reduces the workability of the concrete, impeding the formation of the concrete into the required configuration. Thus, for the following mixing proportion experiments, the moderate W/B of 0.16 was chosen to achieve the designated criterion of high strength on the one hand, and to maintain the workability of the concrete on the other hand.

The high cement content gives rise to the high temperature increase of hydration, as well as autogenous shrinkage of the concrete [45]. To enhance the strength of the concrete and to reduce its autogenous shrinkage [46], two ultrafine powders were used as alternative admixtures to partially replace the cement: silica fume [47], and CCCW [48].

Silica fume is an ultrafine globular active material; its average particle size is 0.1–0.5 μm, which is 100 times smaller than that of the cement. Such fine silica fume can fill the gaps between the cement particles, which can greatly promote the particle packing density effect [49]. A total of 90% of the composition of the silica fume is active silica, which shows strong volcanic activity at room temperature [50]. The silica can react with the calcium hydroxide produced by the hydration of the cement, as shown in Equations (1)–(3) [51], which produces C-S-H gel and further facilitates the hydration process of the cement [52,53]. It can be seen that the strengths show complex variations. The influence of the silica fume is polymorphic. As the silica fume increases, the strengths are increased and exhibit a peak value. They then show significant reductions when the proportion of the silica fume increases to 40%. The lower proportion of the cement reduces the cementation between the aggregates and the particles, which can reduce the homogeneity of the concrete [31].

(1) $${3\;\mathrm{CaO}\cdot \mathrm{SiO}}_{2}{+n\;H}_{2}{O=x\;\mathrm{CaO}\cdot \mathrm{SiO}}_{2}{\cdot y\;H}_{2}O+\left(3-x\right)\;\mathrm{Ca}{\left(\mathrm{OH}\right)}_{2}$$

(2) $${2\;\mathrm{CaO}\cdot \mathrm{SiO}}_{2}{+n\;H}_{2}{O=x\;\mathrm{CaO}\cdot \mathrm{SiO}}_{2}{\cdot y\;H}_{2}O+\left(3-x\right)\;\mathrm{Ca}{\left(\mathrm{OH}\right)}_{2}$$

(3) $${\mathrm{SiO}}_{2}+x\;\mathrm{Ca}{\left(\mathrm{OH}\right)}_{2}{+n\;H}_{2}{O=x\;\mathrm{CaO}\cdot \mathrm{SiO}}_{2}{\cdot y\;H}_{2}O$$

**Figure 9:**
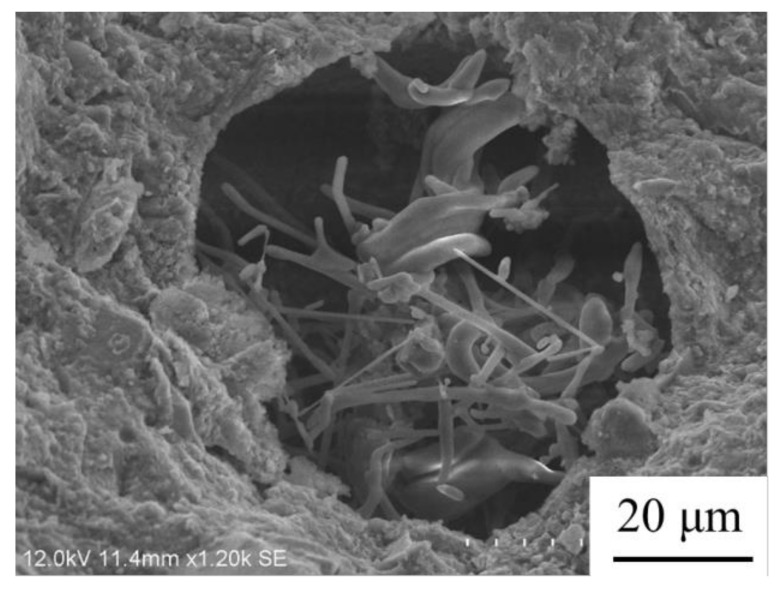
The SEM image of the hydration products of CCCW in USMC.

The Ca^2+^ and SiO_3_^2−^ in the cementitious capillary crystalline waterproof material (CCCW) can react with the unhydrated or partially hydrated cementitious materials and produce tobermorite [54]. The penetration of the CCCW into the gaps and pores of the concrete leads to the reaction of the active compounds in CCCW with the free ions (such as SO_4_^2−^ and AlO^2−^) and compounds (such as CaO) in the concrete, which produces dendritic crystallines (such as hydrated sodium silicate) [55] that are not soluble in water (see Figure 9). Due to the high porosity of coral aggregates, the produced crystallines can fill the pores within the coral, thus improving the densification of the coral aggregates [56]. Increasing the CCCW content raises the compressive strength and flexural strength up to a threshold value of CCCW content, beyond which a further increase in CCCW content, with a corresponding reduction in the cohesiveness of the concrete, lowers the homogeneity and strengths of the concrete.

### 3.2. The Influence of Multiple Combined Binders on Mechanical Properties

The single-factor experiments determine the optimal range of each of the constituent contents of the binder for the enhancement of the mechanical strength of the studied concrete. For this section, the mix designs were performed to find the optimal mix proportion for the development of the ultrahigh-strength concrete. Due to the more diverse and significant effects on the compressive strength, the ordinary Portland cement, silica fume, and CCCW are considered as the main design parameters in the mixing system in the present study. The mass fractions of these three constituents are described in Equation (4). For CCCW, the optimal range of mass fraction was set to 0.1–0.2, while for silica fume it was 0.15–0.25. It should be noted that excessive cement constitution leads to intensive release of the hydration heat, which can result in a severely autogenous shrinkage and generate fine cracks within the concrete [39]. The mass fractions of the cement in the fabricated ultrahigh-strength concretes listed in Table 2 are all higher than 0.73. To reduce the autogenous shrinkage while maintaining the high strength performance of our concrete, a range of mass fractions of 0.55–0.7 was chosen for the cement. The 28-day compressive strength is considered as a design parameter and an optimization criterion.

(4) $$\left\{\begin{array}{c} \begin{array}{c} 0.55\;\leq \;A\;\leq \;0.70\\ 0.10\;\leq \;B\;\leq \;0.20\\ 0.15\;\leq \;C\;\leq \;0.25\\ A+B+C=1 \end{array} \end{array}\right.$$

where A, B, and C represent the fractions of OPC, CCCW, and SF, respectively.

**Figure 10:**
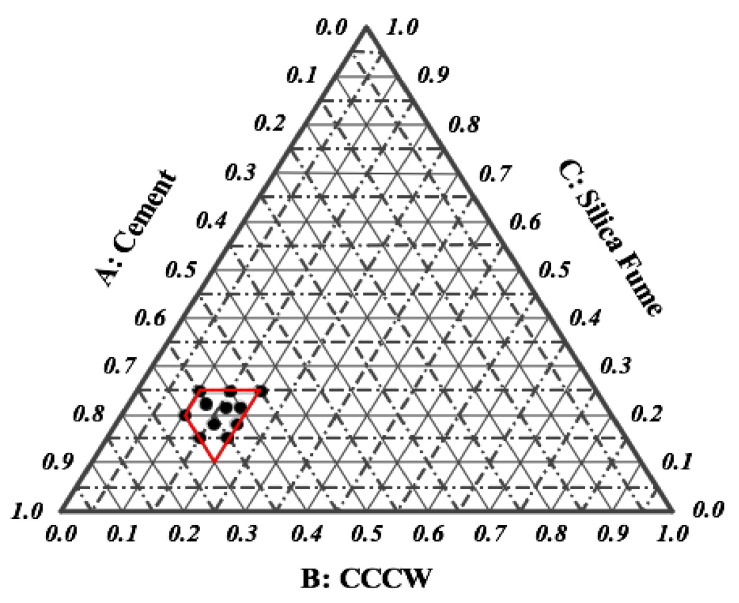
Spectral points of optimal mixture design.

To determine the optimal mix proportions, w built a hybrid regression model and obtained the response surface with the help of the Design-Expert software [57]. The IV-optimal design method was implemented, and 16 mix proportions as well as the consequent compressive strength were determined [58] (see Table 6). It should be noted that there were 5 spectral points located in the same position; thus, only 11 points are presented in Figure 10.

**Figure 11:**
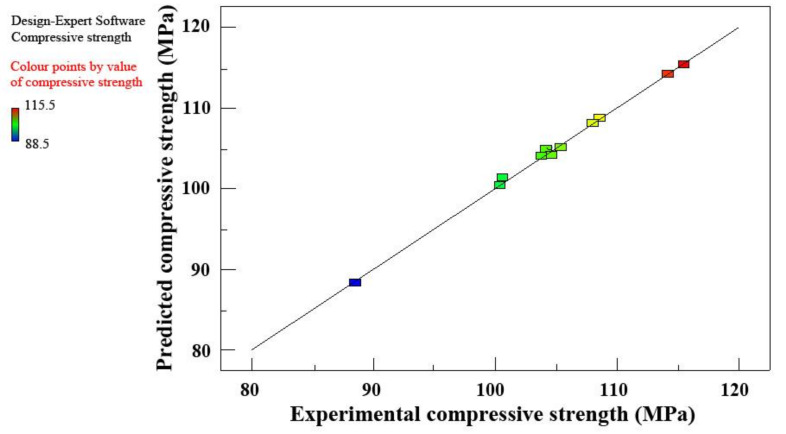
Predicted compressive strength vs. experimental compressive strength.

The regression equation (Equation (5)) is determined by using a select cubic model for the mix designs. The results predicted by the regression equation fit the experimental data very well (Figure 11), indicating that the selected model can successfully identify the correlation between the mixture components.

(5) $$\begin{array}{cc} Y= & 165.65\;A+68.12\;B+85.57\;C\;-\;32.67\;\mathrm{AB}\;-\;101.01\;\mathrm{AC}+45.75\;\mathrm{BC}\\ & +242.01\;\mathrm{ABC}\;-\;210.58\;\mathrm{AB}\left(A-B\right)\;-\;133.35\;\mathrm{AC}\left(A-C\right)\;-\;131.31\;\mathrm{BC}\left(B-C\right) \end{array}$$

The discrepancy between R^2^ and AdjR^2^ is 0.0036, which further indicates the reliability of the cubic model [59]. To quantify the effects of the interaction of parameters and identify the significance of the mix model, analysis of variance was performed [60]; results are given in Table 7. The model F-value of 276.45 implies that the model is significant. P-values of ‘‘Prob > F’’ less than 0.05 indicate that the model terms are significant [61]. In our case, the linear mixture components AC, AB(A−B), AC(A−C), and BC(B−C) are significant model terms.

**Figure 12:**
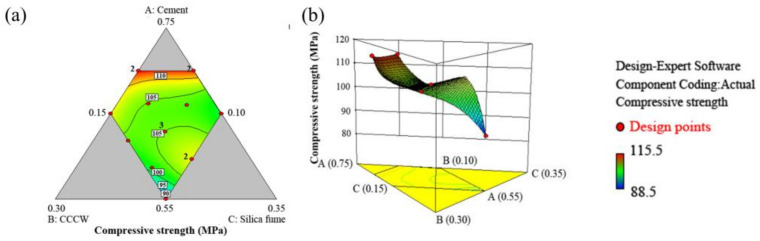
Response surface of the effects of cement, CCCW, and silica fume on the compressive strength: (**a**) contour; (**b**) 3D surface.

The response surface illustrates the effects of the three independent parameters on the compressive strength (see Figure 12). It can be seen that the Portland cement plays the most important role in the enhancement of the compressive strength. Additionally, the contribution of the silica fume is higher than that of the CCCW.

Based on the results calculated by the Design-Expert software, an optimal mix proportion associated with the targeted high compressive strength was attained. To verify the optimized mix proportion [62], the three tests were conducted to estimate the compressive strength. It is notable that the three simultaneously repeated measurements are used to increase the confidence in experimental data and decrease the uncertainty in our estimation. The mix proportions, compressive strength, and flexural strength are given in Table 8; the average compressive strength is 116.76 MPa, which achieves our expectation, and is higher than that of the original mix proportion. The average flexural strength is 18.24 MPa.

### 3.3. Microstructure Characterization and Performance Test of Optimized Concrete

The cement dosage in the original mix proportion was 858 kg/m^3^, while this was decreased by 12.47% (751 kg/m^3^) in the optimal mix proportion. The high compressive strengths of these concretes are attributed to their high binder content, high ultrafine powder content, and low W/B. Due to the low W/B and high content of silica components, the hydration heat of the concrete is relatively high; thus, the internal relative humidity decreases quickly [63]. Such intensive hydration reactions can result in high early-age autogenous shrinkage and shrinkage cracking, which negatively affect the strength development of the concrete [64].

**Figure 13:**
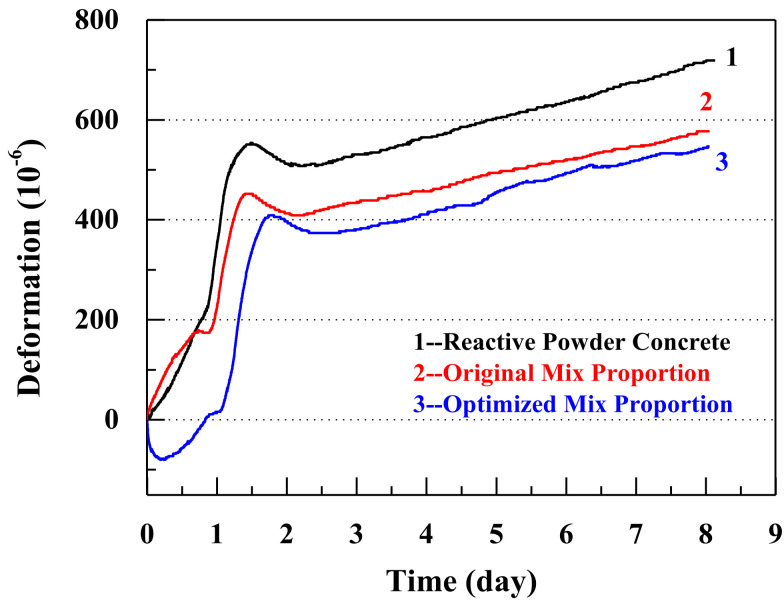
The autogenous shrinkage properties of the three different concretes.

Figure 13 illustrates the autogenous shrinkages of the reactive powder concrete, concrete with the original mix proportions, and concrete with the optimized mix proportions. Compared with the autogenous shrinkages in the reactive powder concrete and in the original mix proportions, the autogenous shrinkage is reduced by 36.89% and 4.65%, respectively, using the optimized mix proportions.

The hydration heat reduces with a decrease in the cement content, which leads to a reduction in the autogenous shrinkage. Additionally, by means of the internal curing within the coral sand, the autogenous shrinkage reduces further. The fine pore structures of the coral sand play a decisive role in the inhibition of the autogenous shrinkage [65].

**Figure 14:**
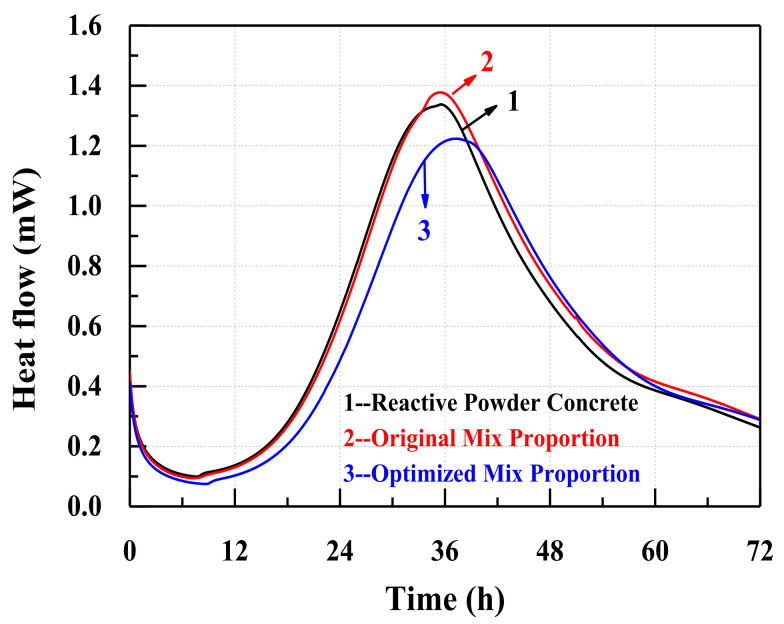
The heat flow test of the three different concretes.

**Figure 15:**
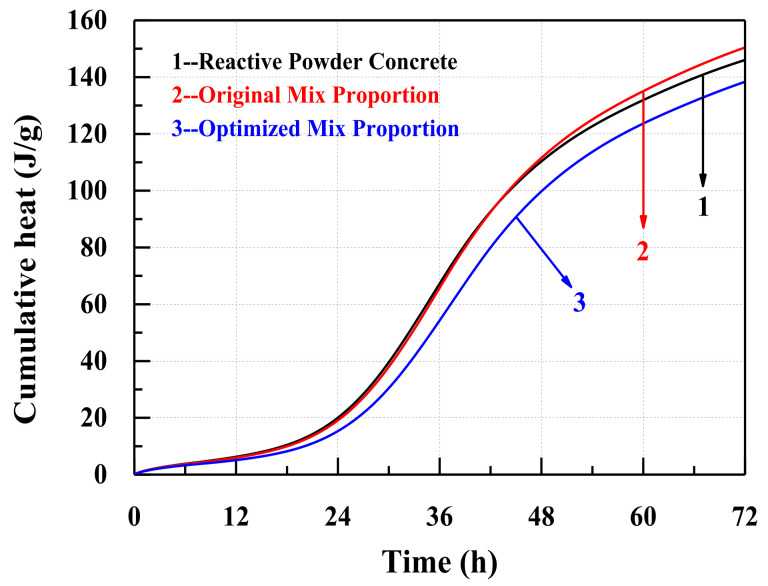
The cumulative heat test of the three different concretes.

The hydration heat flows of samples are depicted in Figure 14. For all three concretes, the hydration heat flow decreases gradually during the first 7 h, and then increases to the maximum value between 30 h and 38 h. The highest heat flow and the cumulative heat of the concrete with the optimized mixture proportions are both lower than those of the reactive powder concrete (in Table 9) and the concrete with the original mix proportions (see Figure 14 and Figure 15). Due to the retardation of the high content of the superplasticizer, the highest heat flow of the concrete with the optimized mixture proportions is maintained for a relatively longer time (Figure 14) [66].

**Figure 16:**
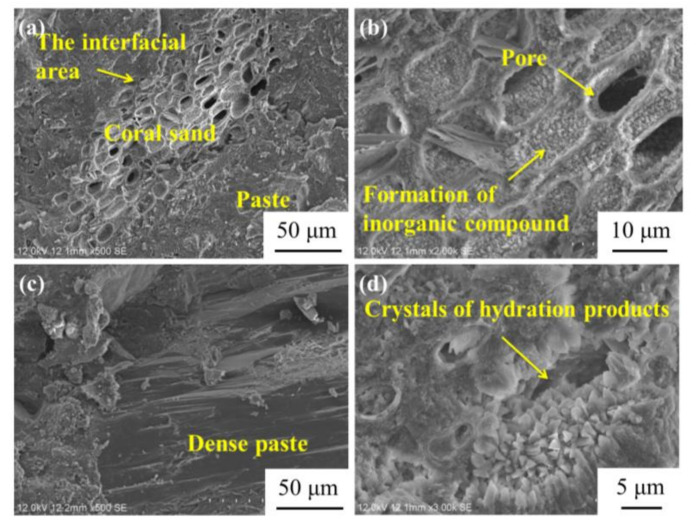
The microstructures of USMC: (**a**) Interface area, (**b**,**c**) Paste area, (**d**) hydration products area.

A scanning electron microscope was employed to investigate the microstructures of the concrete with the optimal mix proportions (see Figure 16). The microstructures of the coral sand, the sample, and the interfacial area can be seen clearly in Figure 16a. Nearly no obvious defects in the interfacial zone between the coral sand and sample are observed [67]. There are numerous pores in the coral sand, while the great majority is filled with the inorganic compounds generated by the reactions within the concrete (see Figure 16b). Judging by the osmotic crystallization of CCCW in the pores, these compounds may be the products of the CCCW hydration. The sample is packed with compact microstructures (Figure 16c). There are nearly no gaps or defects within the sample [68]. The crystals of the hydration products can be seen in Figure 16d. In addition to the Ca(OH)_2_, C-S-H, Aft, and AFm generated by the hydration reaction of the cement, the additive silica fume can also react with Ca(OH)_2_ and produce C-S-H gel [69]. Crystals are produced by the reaction between the CCCW and H_2_O (see Figure 16d).

**Figure 17:**
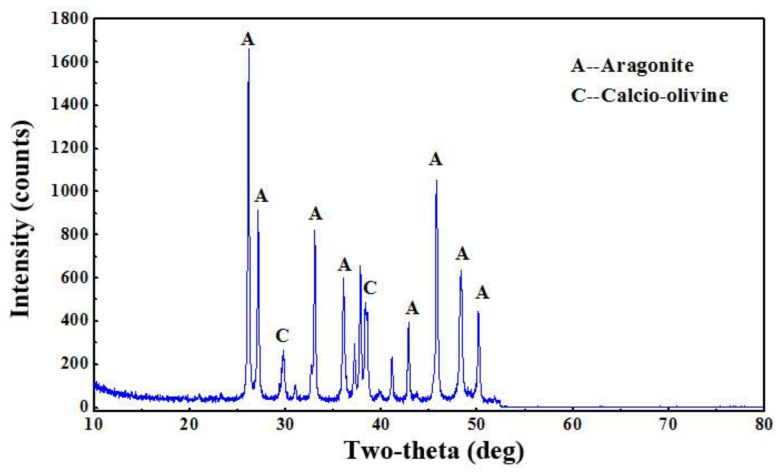
Mineral composition of the coral sand bulk.

**Figure 18:**
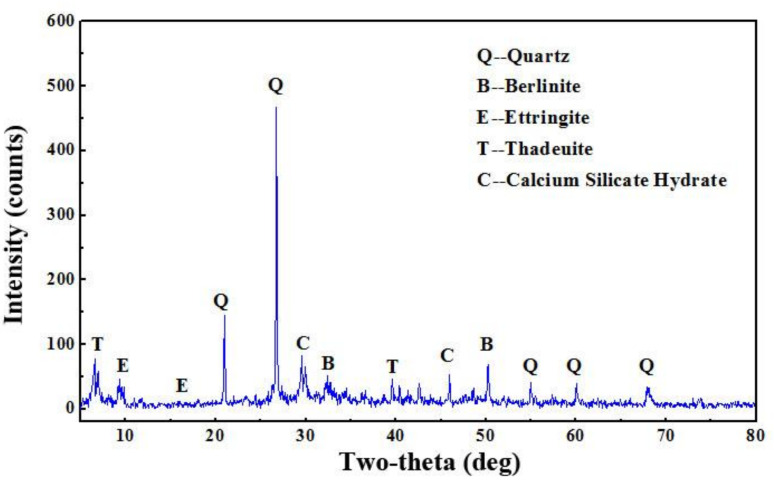
X-ray diffraction pattern of USMC.

The X-ray diffraction of the coral sand and the concrete are shown in Figure 17 and Figure 18, respectively. The aragonite and calcio-olivine are identified in the coral sand (Figure 17). The quartz, berlinite, ettringite, thadeuite, and calcium silicate hydrate are characterized in the concrete (Figure 18). The highest peak position in quartz indicates the siliceous nature of the designed concrete. Quartz fills the pores of the concrete, resulting in the improvement of its compactness [70]. Ettringite and calcium silicate hydrate are the hydration products of the Portland cement and CCCW; they are the main hydration products that contribute significantly to the physical and chemical properties of the concrete [71,72,73]. Berlinite and thadeuite can be the reaction products between calcio-olivine and other compounds, which also fill the pores and reduce the pore size to modify the microstructures and enhance the strengths [49]. The absence of aragonite in the X-ray diffraction of the concrete results from the relatively small fraction of the coral sand in the selected sample. The peak position is too low to be identified.

## 4. Conclusions

An ultrahigh-strength coral aggregate concrete for marine engineering and construction of remote islands has been developed. An average compressive strength of 116.76 MPa and flexural strength of 18.24 MPa are attained by the optimization of the mix proportions for our concrete. The autogenous shrinkage and the heat flow of early hydration reactions of the designed concrete were measured. On account of the lower cement content and the internal curing provided by coral aggregates, the volume change resulting from the autogenous shrinkage was only 63.11% of that of ordinary reactive powder concrete. The highest heat flow and the cumulative heat of the concrete with optimized mixture proportions were both lower than those of the reactive powder concrete and the concrete with the original mix proportions. The microstructures of the main constituents, as well as the interfacial zone between the coral sand and the sample, were characterized via SEM. Our concrete gains flowability and self-compacting ability through the packing of the pores of coral aggregates with hydration products of CCCW. The dense structures observed in the sample and in the interfacial zone facilitate the development of the strength of the designed concrete. The phases formed in our concrete were identified via XRD analysis.

Our present study demonstrates the feasibility of production of the ultrahigh-strength (over 100 MPa) concrete with coral aggregates, and of reducing the autogenous shrinkage through the internal curing provided by coral aggregates.

## Figures and Tables

**Table 1:** Material contents of specimens shown in Figure 1.

Specimen	Binder (kg/m^3^)			Coarse Coral (kg/m^3^)	Coral Sand (kg/m^3^)	Simulated Seawater (kg/m^3^)	SP (kg/m^3^)	W/B
	Cement (P.O 42.5R)	Slag	Fly Ash					
S1 [1]	416	80	72	660	990	160	8	0.3
S2 [1]	606	117	59	361	841	216	16	0.28
S3 [1]	786	151	71	302	705	252	20	0.25
S4 [22]	372			1147 ^NC^	678	228	-	0.6
S5 [23]	450			1020	320	228	21.6	0.5
S6 [24]	440			950	704 ^QS^	176 ^TW^	5.28	0.4

NC: normal carpolite; QS: quartz sand; TW: tap water.

**Table 2:** Chemical properties of raw materials.

	CaO	SiO_2_	Al_2_O_3_	Fe_2_O_3_	MgO	Na_2_O	SO_3_	Loss
Cement (%)	64.13	21.43	2.24	3.78	2.07	0.78	2.25	3.32
Silica fume (%)	0.52	94.51	0.61	0.22	-	-	-	4.14

**Table 3:** Detected material properties of the coral sand.

Detecting Items	Units	Detecting Results	Detecting Items	Units	Detecting Results
Moisture content	%	0.2	EC value	mS/cm	0.140
Organic matter	g/kg	2.77	Total nitrogen	g/kg	0.174
Available phosphorus	mg/kg	6.8	Total phosphorus	g/kg	0.075
pH		9.3	Total potassium	g/kg	0.991
Bulk density	g/m^3^	1.137	Available boron	mg/kg	0.201
Texture		Sand	Cation-exchange capacity	cmol/kg	1.22
Available nitrogen	mg/kg	12.0	Available copper	mg/kg	0.218
Available potassium	mg/kg	7.38	Available zinc	mg/kg	0.073
Carbonate	g/kg	987	Available iron	mg/kg	0.582

**Table 4:** The original mix proportions.

Sample	Binder (Mass Fraction)		Coral Sand ^a^	Coral Powder ^a^	SP ^a^	W/B
	Cement	Silica Fume				
S1	0.8	0.2	0.88	0.35	0.024	0.14

^a^. Fraction by binder mass.

**Table 5:** Mixing proportions of samples.

Sample	Binder (Mass Fraction)			Coral Sand ^a^	Coral Powder ^a^	SP ^a^	W/B
	OPC	SF	CCCW				
S1	0.8	0.2	-	0.88	0.35	0.024	0.14
S2	0.8	0.2	-	0.88	0.35	0.024	0.16
S3	0.8	0.2	-	0.88	0.35	0.024	0.18
S4	0.9	0.1	-	0.88	0.35	0.024	0.16
S5	0.7	0.3	-	0.88	0.35	0.024	0.16
S6	0.6	0.4	-	0.88	0.35	0.024	0.16
S7	0.9	-	0.1	0.88	0.35	0.024	0.16
S8	0.8	-	0.2	0.88	0.35	0.024	0.16
S9	0.7	-	0.3	0.88	0.35	0.024	0.16

^a^. Fraction by binder mass.

**Table 6:** Mix proportions and compressive strengths of optimal mixture design.

Sample	Binder (Mass Fraction)			Coral Sand ^a^	Coral Powder ^a^	SP ^a^	W/B	Y: Compressive Strength (MPa)
	A: OPC	B: CCCW	C: SF					
S1	0.70	0.10	0.20	0.88	0.35	0.024	0.16	114.21
S2	0.60	0.15	0.25	0.88	0.35	0.024	0.16	108.04
S3	0.65	0.1	0.25	0.88	0.35	0.024	0.16	100.42
S4	0.59	0.19	0.22	0.88	0.35	0.024	0.16	100.66
S5	0.63	0.16	0.20	0.88	0.35	0.024	0.16	105.43
S6	0.7	0.15	0.15	0.88	0.35	0.024	0.16	115.51
S7	0.66	0.13	0.21	0.88	0.35	0.024	0.16	103.82
S8	0.55	0.2	0.25	0.88	0.35	0.024	0.16	88.58
S9	0.66	0.16	0.18	0.88	0.35	0.024	0.16	104.22
S10	0.70	0.10	0.20	0.88	0.35	0.024	0.16	114.21
S11	0.62	0.20	0.18	0.88	0.35	0.024	0.16	104.62
S12	0.63	0.16	0.20	0.88	0.35	0.024	0.16	105.43
S13	0.65	0.20	0.15	0.88	0.35	0.024	0.16	108.64
S14	0.63	0.16	0.21	0.88	0.35	0.024	0.16	105.43
S15	0.70	0.15	0.15	0.88	0.35	0.024	0.16	115.51
S16	0.60	0.15	0.25	0.88	0.35	0.024	0.16	108.04

^a^. Fraction by binder mass.

**Table 7:** Analysis of variance results of parameter interaction.

Source	Sum of Squares	df	Mean Square	F-Value	*p*-Value Prob > F
Model	703.45	9	78.16	276.45	<0.0001
Linear mixture	457.24	2	228.62	808.61	<0.0001
AB	0.095	1	0.095	0.34	0.5826
AC	1.76	1	1.76	6.21	0.0471
BC	0.12	1	0.12	0.41	0.5464
ABC	1.5	1	1.5	5.31	0.0608
AB(A−B)	9.69	1	9.69	34.27	0.0011
AC(A−C)	6.92	1	6.92	24.48	0.0026
BC(B−C)	2.71	1	2.71	9.59	0.0212
Residual	1.70	6	0.28	-	-
Lack of Fit	1.70	1	1.70	-	-
Pure error	0.000	5	0.000	-	-
Cor total	705.15	15	-	-	-

**Table 8:** Experimental results of the optimal mix proportions.

Binder (Mass Fraction)			Coral Powder	Coral Sand ^a^	W/B	SP ^a^	Compressive Strength (MPa)	Flexural Strength (MPa)
OPC	CCCW	SF						
0.70	0.15	0.15	0.35	0.88	0.16	0.024	116.68	18.53
0.70	0.15	0.15	0.35	0.88	0.16	0.024	119.34	18.94
0.70	0.15	0.15	0.35	0.88	0.16	0.024	114.27	17.25

^a^. Fraction by binder mass.

**Table 9:** The mix proportions of the reactive powder concrete.

Sample	Binder (Mass Fraction)		Quartz Powder ^a^	Quartz Sand ^a^	W/B	SP ^a^
	Cement	Silica Fume				
Reactive powder concrete	0.8	0.2	0.34	0.88	0.2	0.024

^a^. Fraction by binder mass.

## Data Availability

The data presented in this study are available on request from the corresponding author.
